# Glycosylation of the *Escherichia coli* TibA Self-Associating Autotransporter Influences the Conformation and the Functionality of the Protein

**DOI:** 10.1371/journal.pone.0080739

**Published:** 2013-11-20

**Authors:** Jean-Philippe Côté, Marie-Ève Charbonneau, Michael Mourez

**Affiliations:** Faculté de Médecine Vétérinaire, Université de Montréal, Saint-Hyacinthe, Québec, Canada; Centre National de la Recherche Scientifique, Aix-Marseille Université, France

## Abstract

The self-associating autotransporters (SAATs) are multifunctional secreted proteins of *Escherichia coli*, comprising the AIDA-I, TibA and Ag43 proteins. One of their characteristics is that they can be glycosylated. Glycosylation of AIDA-I and Ag43 have been investigated, but not that of TibA. It is still not clear whether glycosylation of the SAATs affect their structure or their functionality. Therefore, we have looked at the effects of glycosylation on the TibA adhesin/invasin. TibA is glycosylated by TibC, a specific glycosyltransferase, and the two genes are encoded in an operon. In this study, we have found that the glycosylation of TibA is not limited to the extracellular functional domain, as previously observed with AIDA-I and Ag43. We have determined that unglycosylated TibA is not able to promote the adhesion of bacteria on cultured epithelial cell, even though it is still able to promote invasion, biofilm formation and autoaggregation of bacteria. We have purified the glycosylated and unglycosylated forms of TibA, and determined that TibA is less stable when not glycosylated. We finally observed that glycosylation affects the oligomerisation of TibA and that unglycosylated TibA is locked in a conformation that is not suited for adhesion. Our results suggest that the effect of glycosylation on the functionality of TibA is indirect.

## Introduction

Glycosylation is one the most abundant protein modification in eukaryotes and is also an important feature of many bacterial pathogens [1], [2]. Many of the bacterial proteins that are glycosylated are secreted or surface exposed and play a role in the interaction of the bacterium with its environment [3]. In some cases, bacteria harbor a complex glycosylation pathway. For instance, in the general N-glycosylation pathway in *Campylobacter jejuni*, up to 10 proteins are involved in the synthesis and transfer of a heptasaccharide to an asparagine residue of the acceptor polypeptide [4]. There are more than 65 different proteins glycosylated by this pathway in *C. jejuni* [5]. Similarly, complex O-glycosylation pathways also exist in bacteria [6]. These pathways often lead to the modification of pilin or flagellin subunits. Alternatively, there are examples of glycosylation system that are much more simple. These systems are composed of a single glycosyltransferase that glycosylates a single substrate using sugar precursors hijacked from other pathways. The HMW1c glycosyltransferase of *Haemophilus influenzae* is an example of such a simple system: HMW1c uses a sugar precursor from the lipooligosaccharide pathway to glycosylate the HMW1 adhesin [7].

Recently, a new family of these simple O-glycosyltransferases have been described in various proteobacteria [8]. Members of this family include the *Escherichia coli* adhesin associated heptosyltransferase (Aah) and the glycosyltransferase TibC. These two glycosyltransferases function similarly by adding heptoses hijacked from a precursor in the lipopolysaccharide pathway to their substrates, the autotransporters AIDA-I and TibA respectively [9].

Autotransporters are secreted virulence factors of Gram-negative bacteria that possess a modular organization [10], [11]. They are composed of (i) an N-terminal signal sequence for inner membrane translocation through the sec translocon, (ii) an extracellular passenger domain, which is the functional domain of the autotransporter [12] and (iii) a C-terminal domain that forms in the outer membrane a β-barrel plugged by an α-helix. Furthermore, between the passenger domain and the β-barrel, there is often a junction region that is extracellular and helps with the secretion and the folding of the passenger domain [13]. In the case of TibA, an additional proline-rich domain separates the typical junction region from the C-terminal domain.

TibA is a member of a sub-family of autotransporters called the self-associating autotransporters (SAATs) [14]. The Adhesin Involved in Diffuse Adherence (AIDA-I) and the aggregation factor Ag43 are also members of the SAATs family. These proteins can be glycosylated [15], [16] and are multifunctional, as they mediate the autoaggregation of bacteria, the formation of biofilm and the adhesion and invasion of cultured epithelial cells [17], [18], [19], [20]. They also share sequence similarities; notably, a 19-amino acid sequence that is imperfectly repeated in their passenger domain.

The gene coding for TibA is found in an operon with its glycosyltransferase TibC [21]. *aidA* is also encoded in an operon with its glycosyltransferase *aah* [22]. Aah adds heptose residues to serine or threonine in the passenger domain of AIDA-I and can also glycosylate Ag43 and TibA, suggesting that Aah and TibC are structurally and functionally similar. In fact, Aah and TibC are functionally interchangeable [23]. Glycosylation of AIDA-I by Aah is highly heterogenous and no consensus sequence for glycosylation by Aah was found [15]. Instead, it has recently been determined that Aah recognizes a structural motif rather than a sequence [8].

Loss of glycosylation renders AIDA-I or TibA unable to promote adhesion to cultured epithelial cells [15], [24]. However, the consequences of glycosylation on the functionality of AIDA-I are likely to be an indirect effect, since when it is not glycosylated, AIDA-I is poorly expressed at the surface of the bacteria. Indeed, loss of the adhesion properties is due to the inability of unglycosylated AIDA-I to adopt a conformation preventing its degradation. An effect of glycosylation on the conformation of Ag43 was also observed, but Ag43 is not associated to a specific glycosyltransferase [25]. However, in these two studies using AIDA-I and Ag43, the authors could not determine if glycosylation also affected the function of the SAAT as well as its conformation, as it is the case for the trimeric autotransporter EmaA [26]. Thus, it is still unclear how and why glycosylation affects the functionality of SAATs.

TibA is an ideal model to study the glycosylation of the SAATs. TibA is associated with its own glycosyltransferase, unlike Ag43; and the unglycosylated form is well expressed at the cell surface and can be purified, as opposed to AIDA-I. In this study, we determined that glycosylation has an impact on the stability and the conformation of TibA. We showed that, when not glycosylated, TibA is locked in an oligomeric conformation that is not suited for adhesion. Therefore, our study completes previous ones and shows that glycosylation of the SAATs allows the modulation between the different conformations of these proteins.

## Materials and Methods

### Bacterial strains and plasmids

*E. coli* K-12 strain C600 (New England Biolabs) (F^−^ *thr-1 leuB6 thi-1 lacY1 supE44 rfbD1 fhuA21*) was used in this study. Plasmid pTgH allows the expression of a his-tagged TibA and its specific glycosyltransferase, TibC [18]. The His-tag was inserted at the N-terminus of the passenger domain of TibA and does not affect the expression, the glycosylation or the functionality of TibA [18]. Plasmid pTgH C358R was derived from pTgH and contains a mutation at position 358 in TibC that inactivates the enzyme [23]. Plasmid pTngH allows the expression of his-tagged TibA only and was constructed by site-directed deletion of *tibC* from the plasmid pTgH using the primer ΔtibC (5′-GAATTGGAGCGGATAACAATTTCACACAGGAGTAAGCAATGAATAAGGTC-3′) and its complement using the Quickchange mutagenesis kit (Stratagene).

### Bacterial and mammalian cell growth conditions

Bacteria containing the different plasmids were grown on Luria-Bertani (LB) agar plates or in liquid LB medium containing 100 µg*mL^−1^ ampicillin. Bacterial cultures were grown at 30°C and growth was monitored by measuring the optical density at 600 nm (OD_600_). At an OD_600_ of 0.8, the cultures were induced with 10 µM isopropyl-β-D-thiogalactopyranoside (IPTG) and growth was pursued overnight. This low concentration of IPTG was used to limit the toxicity associated with overexpression of TibA and TibC. HEp-2 cells (ATCC CCL-23) were grown at 37°C with 5% CO_2_ in Dulbecco's modified Eagle's medium (Gibco) containing 10 mM sodium pyruvate (Sigma), 10% bovine growth serum (HyClone), 2.5 µg*mL^−1^ amphotericin B (Fungizone), and 100 µg*mL^−1^ penicillin/streptomycin (Gibco).

### SDS-PAGE and Immunoblotting

To prepare whole-cell extracts, overnight cultures (5 mL) were grown, normalized, centrifuged for 10 min at 12,000X *g* in microcentrifuge tubes, and the pellets were resuspended in 50 µL of Tris-buffered saline (TBS; 50 mM Tris-HCl pH 8 and 150 mM NaCl). Alternatively, outer membrane extracts were prepared as previously described [27]. Bacterial cultures were normalized, harvested and resuspended in 1 mL of Tris-HCl 10 mM pH 8, 0.75 M sucrose, 0.5 mg/mL lysozyme and 10 mM EDTA. The resulting spheroplasts were centrifuged, resuspended in TBS containing a protease inhibitor cocktail (Complete mini; Roche) and lysed by sonication. Cellular debris were removed by a low speed centrifugation (3 min/3000 rpm) and the lysates were further centrifuged at 13,000 rpm for 30 min at 4°C. The pellets containing the membranes were then resuspended in TBS 1% Triton X-100, incubated for 1 h and centrifuged at 13,000 rpm for 30 min at 4°C. The insoluble fractions corresponding to the outer membrane extracts were resuspended in 50 µL of TBS.

The samples were then diluted in twice concentrated SDS-PAGE loading buffer containing β-mercaptoethanol, denatured by heating at 100°C for 10 min and separated by SDS-PAGE on 10% acrylamide gels. The gels were either stained with Coomassie blue or transferred to polyvinylidene fluoride membranes (Millipore). Immunodetection was performed with a serum raised against glycosylated AIDA-I and cross-reacting with glycosylated TibA diluted 1∶50,000 in blocking buffer (5% skim milk, 50 mM Tris-HCl pH 7.5, 150 mM NaCl, 0.05% Triton X-100). Alternatively, a His-tag specific antibody (Invitrogen) was used. Horseradish peroxidase-conjugated secondary antibodies (Sigma) were used according to the instructions of the manufacturer. Immune complexes were revealed using a 3,3′,5,5′-tetramethylbenzidine solution for membranes (Sigma). Bands were quantified by densitometry using the ImageJ software [28] and were compared to glycosylated TibA.

### Protein purification

To purify glycosylated TibA or unglycosylated TibA, one liter of *E. coli* C600 bearing the plasmid pTgH or pTngH, respectively, were grown to an OD_600_ of 0.8 and induced with 10 µM IPTG overnight. Bacteria were harvested and resuspended in 50 mL TBS containing a protease inhibitor cocktail (complete; Roche). Cells were lysed using a French press and an ultrasonicator. Lysates were recovered by a slow speed centrifugation to clear cellular debris and were further centrifuged for 30 min at 250,000X *g*. The pellets, containing the membranes, were then resuspended in 25 mL TBS containing 2% Triton X-100 reduced (Sigma) and 10 mM EDTA. The suspensions were incubated for 30 min at 37°C and centrifuged again for 30 min at 250,000X *g*. EDTA was removed from the supernatant using PD-10 columns (GE Healthcare) and His-tagged TibA was purified by affinity chromatography using a 1 mL His-Trap HP column (GE Healthcare). Purity of the purified proteins was consistently over 95% as evaluated by Coomassie blue stained SDS-PAGE. Finally, the protein samples were dialyzed to replace the Triton X-100 with 1% n-octyl-β-D-glucoside (Roche).

### Mass Spectrometry

Glycosylated his-tagged TibA was purified from *E coli* C600 as described above and run on an SDS-PAGE 10% acrylamide gel. The protein band corresponding to TibA was cut from the gel and destained with water-sodium bicarbonate buffer and acetonitrile. The protein was reduced with di-thiothreitol and alkylated with iodoacetamide prior to in-gel digestion with trypsin. The tryptic peptides were eluted from the gel with acetonitrile containing 0.1% trifluoroacetic acid. The tryptic peptides were then separated on an Agilent Nanopump system using a C18 ZORBAX trap and a SB-C18 ZORBAX 300 reversed-phase column (Agilent Technologies, Inc.) (150 mm by 75 µm; 3.5 µm particle size). All mass spectra were recorded on a hybrid linear ion trap-triple quadrupole mass spectrometer (Q-Trap; AB Applied Biosystems) equipped with a nanoelectrospray ionization source. The accumulation of MS-MS data was performed with Analyst software, version 1.4 (AB Applied Biosystems). MASCOT software (Matrix Science, London, United Kingdom) was used to create peak lists from MS and MS-MS raw data.

### Functional assays

Autoaggregation, biofilm formation, adhesion and invasion assays were performed as previously described [17]. Briefly, *E. coli* C600 bearing an empty vector, the plasmid pTgH or the plasmid pTngH were induced at an OD_600_ of 0.8 and grown overnight in LB or M9 (Biofilm formation) media. In the autoaggregation assay, the overnight cultures were normalized in 5 mL of LB to an OD_600_ of approximately 1.5 in culture tubes and left standing at 4°C. Samples (100 µL) were taken 1 cm below the surface at the beginning of the assay and after 120 min, and the OD_600_ of the samples were measured. In the biofilm formation assay, the overnight cultures were normalized in M9 minimal medium, grown for 24 hours at 30°C in plastic microtiter plates and the biofilms were stained with crystal violet. After washes, the dye was then solubilized with a mixture of ethanol and acetone (80∶20), and the absorption at 595 nm of the solution was measured. In the adhesion and invasion assays, the overnight cultures were inoculated onto monolayers of confluent HEp-2 cells in a 24-well plate (approximately 2.5×10^5^ cells) using 10^6^ colony-forming units (CFU) per well. For adhesion, after 3 h, the cells were washed with PBS, and the adhering bacteria were recovered with 100 µL of Triton X-100 (1%), plated, and counted. For invasion, fresh medium containing 100 µg*mL^−1^ gentamicin was added after the 3 h incubation and washing, and the plates were incubated for an additional 2 h before recovering and plating.

All functional assays were performed at least three times in duplicate or triplicate. For each assay, the results were compared to those of glycosylated TibA by performing an ANOVA and Dunnett post-tests using Prism 4.0 software (Graphpad Software).

### β-Galactosidase reporter assay

The β-galactosidase activity of strains SR1458 [29] and SR1364 [30] transformed with an empty vector, plasmid pTgH, or plasmid pTgHC358R was assessed as described previously [30], and the results were expressed in Miller units. Statistical comparisons were performed by ANOVA using Prism 4.0 (GraphPad software).

### Proteases accessibility assay

Overnight cultures of strain C600 bearing an empty vector, the plasmid pTgH or the plasmid pTngH were normalized and resuspended in TBS in the presence or absence of trypsin (Sigma) or proteinase K (Invitrogen) at a final concentration of 0.5 µg/mL, 1 µg/mL or 5 µg/mL. After 30 min of incubation on ice, the samples were diluted in twice concentrated SDS-PAGE loading buffer containing β-mercaptoethanol, denatured by heating at 100°C for 10 min, separated by SDS-PAGE and visualized by immunoblotting using the anti-His antibody.

### Far-UV Circular Dichroism

Far-UV CD spectra of the proteins were recorded on a spectropolarimeter (Jasco Spectroscopic Co. Ltd.; model J-810) using a 0.1-cm-path length cuvette. The far-UV CD spectra of glycosylated and unglycosylated TibA (500 µg/mL in TBS 1% n-octyl-β-D-glucoside) were recorded between 205 and 260 nm. For each spectrum, ten accumulations were averaged, and the contribution of buffer to the measured ellipticity was subtracted. To study the effect of salts, the protein samples were diluted prior to measurement in TBS containing NaCl in order to give a final NaCl concentration of 500 mM and a final detergent concentration of 0.2–0.3% which is below the critical micelle concentration of n-octyl-β-D-glucoside. To study the effect of temperature, the ellipticity of the proteins was recorded at 218 nm with the temperature varying between 20°C and 80°C at a rate of 5°C/min. Ellipticities were converted to mean residual ellipticities (MRE).

## Results

### Glycosylation is not required for optimal expression of TibA

**Figure 1:**
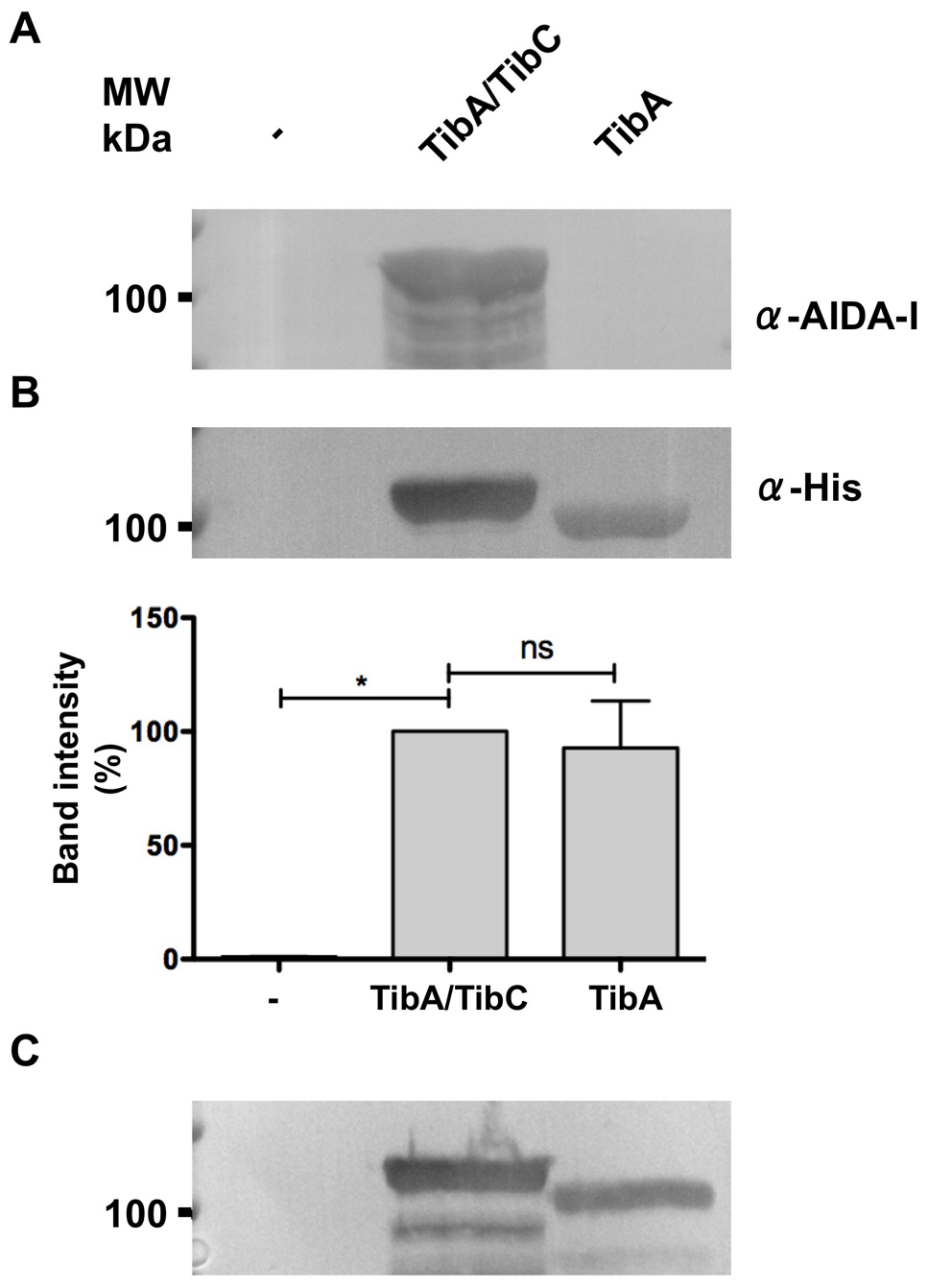
Effect of glycosylation on the expression level of TibA. Whole cell extracts of *E. coli* strain C600 bearing an empty vector (-), or a plasmid allowing the expression of glycosylated TibA (TibA/TibC) or unglycosylated TibA (TibA) were separated by SDS-PAGE and revealed by immunoblotting with an anti-AIDA-I antibody (A) or an anti-His antibody (B). The intensity of the bands probed with the α-His antibody was quantified using ImageJ and values were normalized to the amount of glycosylated TibA (lower panel). Experiments were done five times and ANOVA and Dunnett post-tests were used to identify significant (*; p<0.05) and non-significant (ns) differences with glycosylated TibA. (C) Outer membrane extracts of *E. coli* strain C600 bearing an empty vector (-), or a plasmid allowing the expression of glycosylated TibA (TibA/TibC) or unglycosylated TibA (TibA) were revealed by immunoblotting using the anti-His antibody.

Glycosylation is often important for protein stability [31]. This is the case for another SAAT, AIDA-I. The lack of glycosylation of AIDA-I results in a significant decrease in protein expression [15]. Therefore, we first determined the effect of glycosylation on the expression levels of TibA. To produce glycosylated TibA, we used a plasmid which harbors genes coding for an His-tagged TibA and TibC [18]. To produce unglycosylated TibA, we used a plasmid that expressed His-tagged TibA only. Whole-cell extracts of *E. coli* strain C600 bearing an empty vector, or plasmids allowing the production of glycosylated or unglycosylated TibA were probed with an antibody raised against the His-tag (Fig.1B). The results showed that the expression level of TibA was similar whether it was glycosylated or not.

The whole cell extracts were also probed with an antibody raised against glycosylated AIDA-I (Fig. 1A). The anti-AIDA-I antibody recognizes AIDA-I only when it is glycosylated [9] and cross-reacts with glycosylated TibA [18]. This confirmed that TibA expressed with TibC was glycosylated while TibA expressed alone was not. We have also expressed unglycosylated TibA from a plasmid coding for a mutated and inactive TibC glycosyltransferase [23]. Unglycosylated TibA was also expressed at a similar level than glycosylated TibA when produced from this plasmid (data not shown).

Lastly, we confirmed that unglycosylated TibA is localized in the outer membrane. Outer membrane protein extracts of *E. coli* bearing an empty vector, or plasmids allowing the production of glycosylated or unglycosylated TibA were probed using the anti-His antibody (Fig. 1C). The results showed that both forms of the protein are present in the outer membrane of *E. coli*. Taken together, our results suggest that, unlike for AIDA-I, the lack of glycosylation does not influence the expression of TibA and its insertion in the outer membrane.

### Localization of the glycosylation sites in TibA

We next sought to determine the regions of TibA that were glycosylated. We purified glycosylated his-tagged TibA by affinity chromatography from membrane extracts of *E. coli* strain C600 expressing glycosylated TibA. After SDS-PAGE, the band corresponding to TibA was digested by trypsin within the gel and the resulting peptides were analyzed by electrospray tandem MS/MS [15]. TibA is glycosylated by heptose residues [23]. Therefore, a peptide modified by one or more heptose residues harbors an increase of its mass by a multiple of 192 Da.

**Figure 2:**
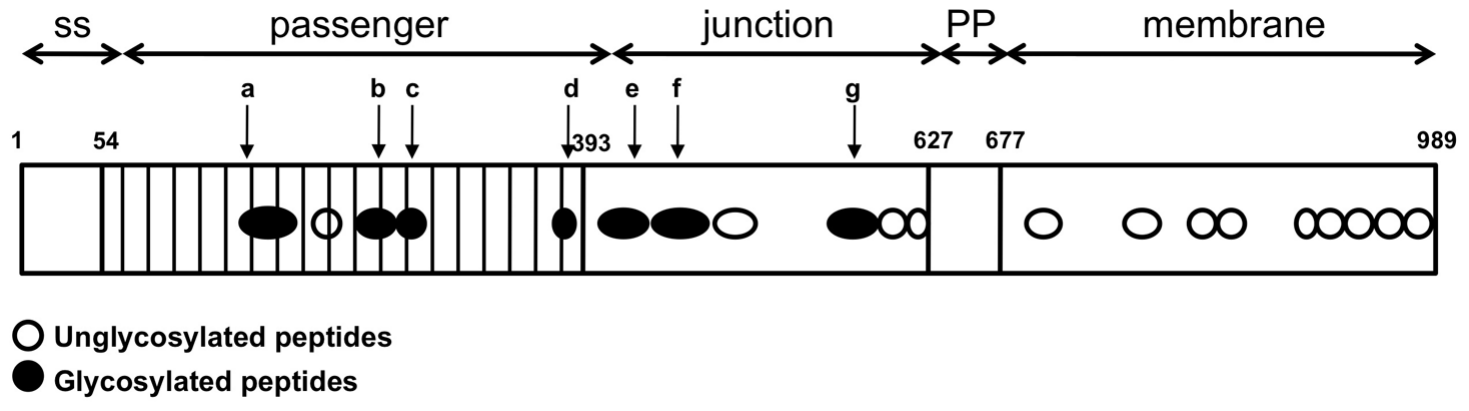
Localization of the peptides in TibA. Glycosylated (filled circle) and unglycosylated (empty circle) peptides from Table 1 are localized on a schematic representation, drawn to scale, of TibA highlighting the signal sequence (residues 1 to 54), the passenger domain (residues 54 to 393), the junction region (residues 393 to 627), the proline-rich domain (residues 627 to 677) and the membrane-embedded domain (residues 677 to 989).

Twenty peptides were identified throughout the entire protein and represent 47% of the protein (Fig. 2 and Table 1). Seven of these peptides were found to be glycosylated: their mass presented one to seven addition of 192 Da compared to the theoretical mass. Four of the seven peptides were located in the passenger domain. The remaining three-glycosylated peptides were located in the junction region, showing that glycosylation is not limited to the passenger domain of TibA. As is the case for AIDA-I [15] and Ag43 [25], glycosylation of TibA was heterogenous; one peptide could be found with various amounts of heptose residues. Unfortunately, we could not determine by this technique onto which amino acid the heptoses were added and, in the case of a peptide modified with multiple heptoses, if the heptoses were on the same residue or on different residues. However, the glycosylated peptides were rich in serines and threonines and did not contain N-glycosylation motifs. Thus, the heptoses were most likely added by O-glycosylation, as it is the case for AIDA-I and Ag43 [15], [25].

**Table 1:** Glycosylated and unglycosylated peptides in TibA.

Glycosylated peptides					
	Sequence	Theoritical mass	# of heptoses	Observed mass	Score^a^
**a**	Q_147_ TVFSGGSAMGTIINSGGDQYVISGGSATSASVTSGAR_184_	3579.85	5	4538.97	52
			6	4730.09	45
			7	4923.64	66
**b**	T_234_ TINSGGGMYLYGGSATGTSIYNGGR_260_	2555.76	1	2747.65	37
			2	2939.51	39
**c**	Q_261_ YVSSGGSATNTTVYSGGR_279_	1891.97	3	2468.36	35
**d**	S_376_ GGVLYGTTTLTDK_389_	1412.56	0	1411.74	53
			1	1603.65	55
**e**	F_432_ SGLLSQDGGIFLQSGGAMTMDALQAK_458_	2744.13	1	2935.29	68
**f**	A_459_ NVTTQSGTTLTLDNGTILTGNVAGDSTGAGDMAVK_494_	3452.75	6	4604.48	36
**g**	N_576_ TGLEPVSAGAPLQVVQTGGGDAAFTLK_603_	2699.01	0	2697.07	36
			1	2889.65	75

Unglycosylated peptides				
Q_204_ YVRDGGSATDTVLNNTGR_222_	2024.13		2023.23	43
G_495_ ASVWHLDGDSTVGALTLDNGTVDFRPSTTTR_526_	3347.60		3346.64	47
G_604_ GKVDAGTWEYGLSK_618_	1567.72		1566.80	66
E_619_ NTNWYLK_627_	1067.17		1067.40	39
S_689_ VDAVLGMATAPAYVFNSELDNLR_712_	2553.87		2553.26	56
S_790_ NVDSSGGGLYATWFDNDGYYVDGVLK_816_	2900.06		2899.47	66
G_836_ DYSQNGFGGSLEAGR_851_	1614.65		1613.94	92
T_852_ FSLNENAWAQPYVR_866_	1795.97		1795.79	93
L_901_ GMTLDVAGK_909_	1004.21		1004.61	55
E_910_ VKPYLSAAVSHEFSDNNK_929_	2135.32		2135.18	65
I_932_ NDTYDFRNDISGTTGK_948_	1917.02		1917.25	35
Y_949_ GLGVNAQLTPNAGVWAEAR_968_	2087.32		2086.94	92
Y_969_ ENGKQTESPITGGVGFR_986_	1940.10		1940.12	62

a A score >35 indicate identity or extensive homology (probability of a random match, p<0.05).

### Glycosylation of TibA is required for adhesion

**Figure 3:**
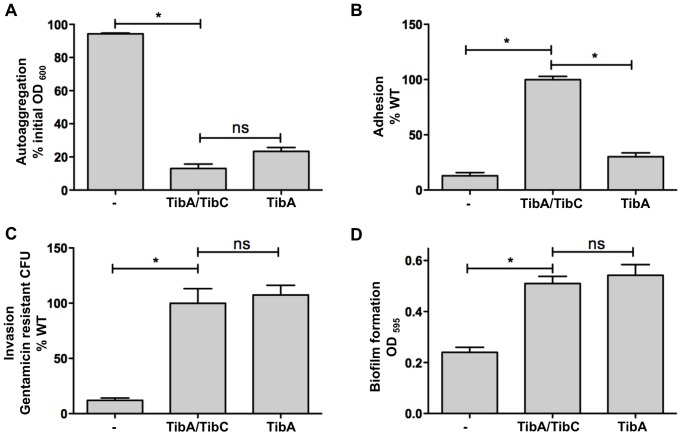
Effect of glycosylation on the functionality of TibA. (A) Autoaggregation assay: *E. coli* C600 bearing an empty vector (-), a plasmid allowing the expression of glycosylated TibA (TibA/TibC) or unglycosylated TibA (TibA) were normalized to an OD_600_ of 1.5 and left standing. OD_600_ at the top of the culture was measured at the beginning of the assay and after 2 h. Results are shown in percentage of initial OD_600_. (B) Adhesion assay: Bacteria were inoculated onto monolayers of confluent Hep-2 cells. After 3 h, the adhering bacteria were recovered, plated and counted. Results represent the percentage of adhered bacteria compared to glycosylated TibA. (C) Invasion assay: After adhesion, extracellular bacteria were killed by addition of gentamicin and invaded bacteria were recovered, plated and counted. Results represent the percentage of gentamicin resistant bacteria compared to glycosylated TibA. (D) Biofilm formation assay: Biofilms were stained with crystal violet and the amount of fixed dye was determined by measuring OD_595_. Experiments were done three times in duplicate and ANOVA and Dunnett post-tests were used to identify significant (*; p<0.05) and non-significant (ns) differences with glycosylated TibA.

To determine the effect of glycosylation on the functionality of TibA, we have performed autoaggregation, adhesion, invasion and biofilm formation assays with *E. coli* C600 bearing an empty vector, or plasmids allowing the expression of TibA and TibC or TibA alone (Fig. 3). The results were similar when unglycosylated TibA was produced by the plasmid allowing the expression of TibA and the mutated and inactive TibC (data not shown). Bacteria expressing unglycosylated TibA were able to autoaggregate, form biofilm and invade cultured epithelial cells as well as the bacteria expressing glycosylated TibA, but were impaired in adhesion. Mutants that can invade but are not able to adhere were also isolated in structure-function studies of TibA and AIDA-I [17], [18], suggesting that adhesion and invasion are not linked together and rely on distinct mechanism. These results suggest that glycosylation might play a role in the adhesion mediated by TibA.

### Unglycosylated TibA causes an extracellular stress

**Figure 4:**
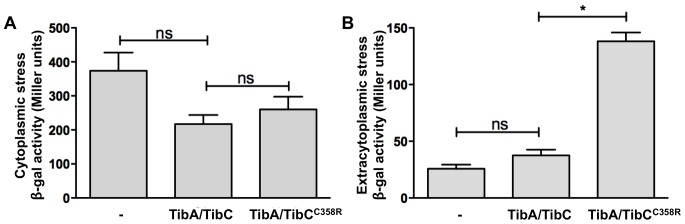
Effect of glycosylation on the generation of cytoplasmic and extracytoplasmic stress. Reporter *E. coli* strains SR1364 (A) and SR1458 (B) were transformed with an empty vector (-), a plasmid allowing the expression of glycosylated TibA (TibA/TibC) or unglycosylated TibA (TibA/TibC^C358R^) and the β-galactosidase activity was measured. Experiments were done three times in duplicate and ANOVA and Dunnett post-tests were used to identify significant (*; p<0.05) and non-significant (ns) differences with glycosylated TibA.

Proteins bearing an abnormal conformation are sensed by specialized stress-sensing system, which leads to the expression of chaperones and proteases in order to correct the situation [32]. To determine the impact of glycosylation on the conformation of TibA, we monitored the cellular stress generated by the expression of glycosylated and unglycosylated TibA using two previously described reporter strains. In strain SR1364, β-galactosidase activity is under the control of the *rpoH* promoter that is induced by a cytoplasmic stress [30]. In strain SR1458, it is under the control of the *degP* promoter, which is induced by an extracytoplasmic stress [29]. Therefore, a cytoplasmic stress will lead to β-galactosidase activity in strain 1364, while an extracytoplasmic stress will lead to β-galactosidase activity in strain 1458. We have allowed the expression of glycosylated or unglycosylated TibA in strains SR1364 and SR1458 and we have monitored the β-galactosidase activity upon expression of TibA. In this experiment, unglycosylated TibA was produced using the pTgH C358R that produce an inactive TibC glycosyltransferase. Glycosylated TibA did not induce the β-galactosidase activity in either strain, suggesting that it does not induce any cytoplasmic or extracytoplasmic stress (Fig. 4). However, unglycosylated TibA induced β-galactosidase activity in strain SR1458, meaning that it induces extracytoplasmic stress and suggesting that it adopts an abnormal conformation. This suggests that unglycosylated and glycosylated TibA harbor different conformations when they reach the extracellular compartment in *E. coli*.

### Glycosylation does not affect the final conformation of TibA

**Figure 5:**
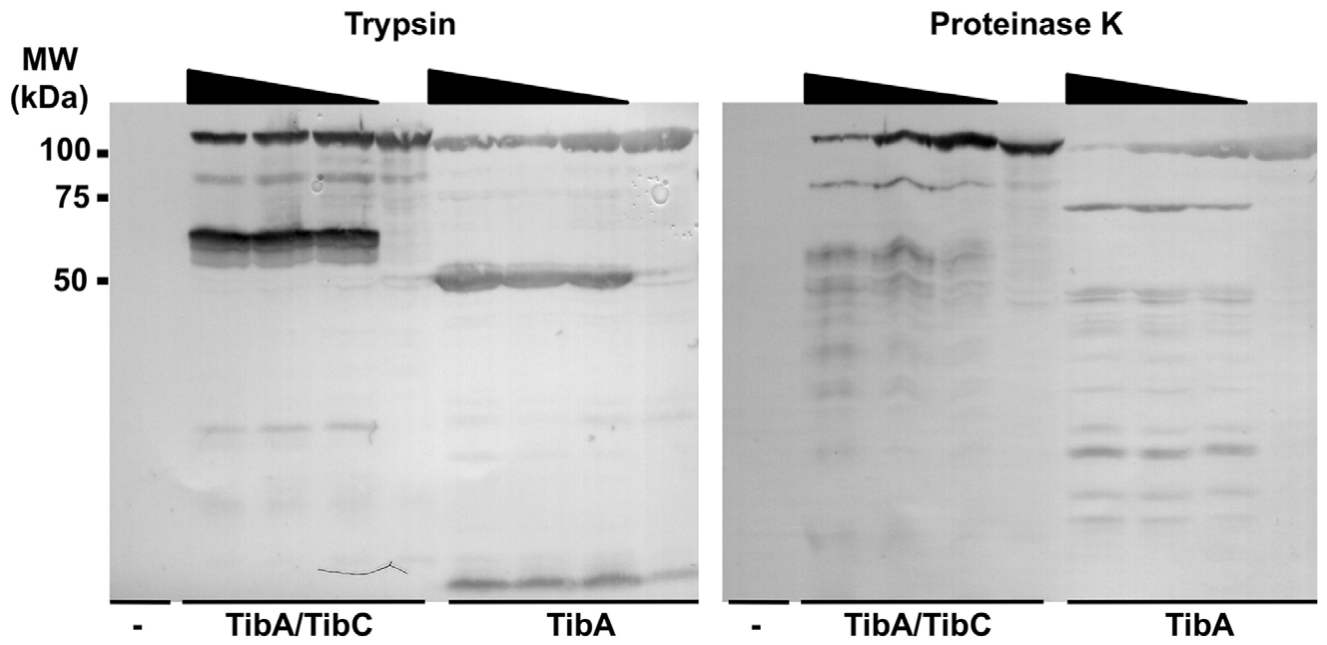
Effect of glycosylation on protease susceptibility. *E. coli* strain C600 bearing an empty vector (-), or a plasmid allowing the expression of glycosylated TibA (TibA/TibC) or unglycosylated TibA (TibA) were pelleted and resuspended in TBS in the presence or absence of trypsin or proteinase K (0.5 µg/ml, 1 µg/mL and 5 µg/mL). After 30 min, whole cell extracts were prepared and revealed by immunoblotting with an anti-His antibody. MW, molecular weight.

Proteins harboring an aberrant conformation are usually more sensitive to proteases [33], [34]. Therefore, we have performed limited proteolysis of the bacterial surface proteins in order to determine if unglycosylated TibA is more sensitive to proteolytic degradation than its glycosylated counterpart. We have subjected *E. coli* strain C600 expressing glycosylated TibA or unglycosylated TibA to an increasing amount of trypsin or proteinase K (Fig. 5). We observed that the glycosylation did not affect the sensitivity of TibA to either trypsin or proteinase K. Both proteins were degraded by the same amount of proteases and showed a similar profile of degradation. Since TibA is as well expressed whether it is glycosylated or not, it is possible to purify the unglycosylated form, as opposed to AIDA-I [15]. Therefore, we also performed limited proteolysis on the purified proteins. Again, both proteins showed a similar sensitivity to trypsin (data not shown). Thus, we conclude the final conformations of glycosylated and unglycosylated TibA, as probed by differences in proteolysis sensitivity, do not seem to be different.

**Figure 6:**
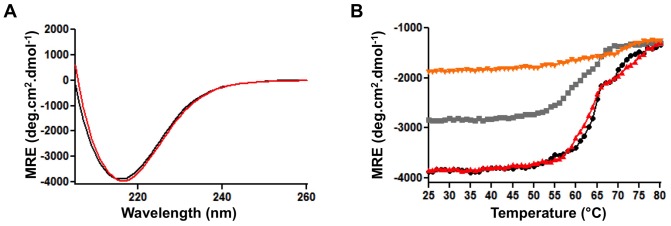
Effect of glycosylation on purified TibA. TibA was purified from *E. coli* strain C600 bearing plasmids allowing expression of glycosylated and unglycosylated TibA and solubilized in TBS 1% n-octyl-β-D-glucoside. (A) Far-UV CD spectrum of glycosylated TibA (black) and unglycosylated TibA (red) are shown between 205 and 260 nm. (B) Proteins were also subjected to a thermal denaturation followed by a renaturation monitored by far-UV CD. Temperature was increased from 25°C and 80°C at a rate of 5°C per minute and the ellipticities at 218 nm were recorded for glycosylated TibA (black) and unglycosylated TibA (red). Right after the denaturation, temperature was decreased from 80°C to 25°C at a rate of 5°C per minute and the ellipticities were recorded at 218 nm for glycosylated TibA (gray) and unglycosylated TibA (orange).

We next monitored the conformations of the purified proteins by far-UV circular dichroism. The CD spectra of glycosylated TibA and unglycosylated TibA were similar (Fig. 6A) and showed a single minimum of ellipticity at 218 nm, which is characteristic of β-stranded proteins. This is in agreement with the predicted structure of TibA [35]. This result again suggests that unglycosylated TibA harbors a similar final conformation than glycosylated TibA. To measure the stability of the protein, we followed the thermal denaturation of glycosylated and unglycosylated TibA by measuring the ellipticity at 218 nm. For both glycosylated and unglycosylated TibA, the denaturation followed a simple two-state model with a denaturation temperature (T_m_) of ∼65°C (Fig 6B). Right after denaturation, we allowed the protein to refold by decreasing the temperature. We observed that glycosylated TibA was able to refold to a certain extent, but unglycosylated TibA did not refold at all. This suggests that glycosylation affects the dynamics of TibA folding, rather than its final conformation.

### Glycosylation allows the modulation between the different conformations of TibA

In the case of AIDA-I, the conformation and oligomerisation of the protein can be modulated by environmental signals, such as bile salts [36]. Furthermore, this change is associated with the functionality of the protein: signals that modulate the conformation and oligomerisation of AIDA-I also modulate AIDA-mediated autoaggregation of bacteria. One such signal is high concentrations of sodium chloride. Since glycosylation seemed to affect the dynamics of TibA folding, we tested the effect of glycosylation on salt-induced conformational changes in TibA.

**Figure 7:**
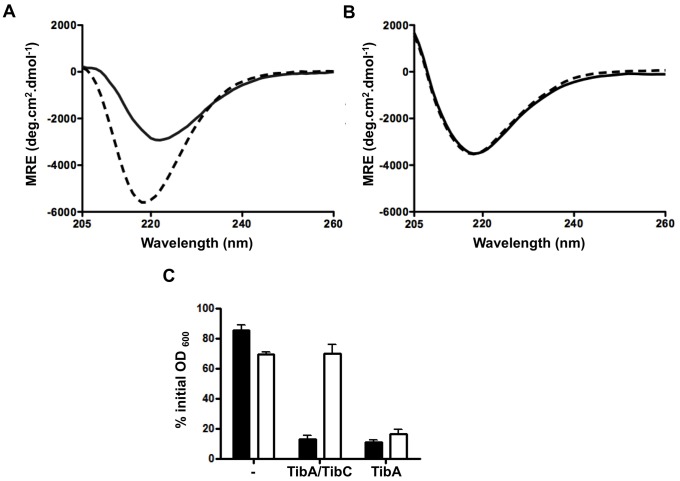
Effect of glycosylation on the modulation of conformation by salt. Far-UV CD spectrum of glycosylated TibA (A) and unglycosylated TibA (B) are shown between 205 and 260 nm. The purified proteins were solubilized in TBS 1% n-octyl-β-D-glucoside (bOG). Prior to CD measurement, the proteins were diluted to a final bOG concentration of 0.3%, below the CMC, in TBS (black line) or in TBS 500 mM NaCl (dotted line). (C) *E. coli* strain C600 bearing an empty vector (-), or a plasmid allowing the expression of glycosylated TibA (TibA/TibC) or unglycosylated TibA (TibA) were resuspended in TBS (black bars) or in TBS 500 mM NaCl (white bars) and autoaggregation assays were performed.

Previous experiments were realized in the presence of detergent on monomers of TibA because detergent inhibits the oligomerisation of the purified protein (data not shown). As a result, in order to observe the effect of glycosylation on the oligomerisation of TibA, we assessed the conformation of purified glycosylated and unglycosylated TibA in the presence NaCl and in the absence of detergent. To trigger oligomerisation, we diluted the protein sample below the critical micelle concentration (CMC) of n-octyl-β-D-glucoside in the presence or absence of 500 mM NaCl. We observed that addition of salt to glycosylated TibA resulted in a decrease of the ellipticity and a blue-shift in the minimum of ellipticity; two characteristics highlighting conformational changes in the protein (Fig 7A). Furthermore, the addition of salt inhibited the autoaggregation of bacteria expressing glycosylated TibA (Fig. 7C). This suggests that TibA can be found in two different conformations, as is the case for AIDA-I: one conformation that mediates oligomerisation of the protein and autoaggregation of the bacteria and the other that is not competent for oligomerisation and autoaggregation. Interestingly, the addition of salt did not modify the CD spectrum of unglycosylated TibA and did not inhibit the autoaggregation of bacteria expressing unglycosylated TibA (Fig. 7B–C). Thus, when not glycosylated, TibA is only found in the conformation that is competent for oligomerisation and autoaggregation. This is consistent with the hypothesis that glycosylation of TibA is important for the modulation of its conformation.

## Discussion

In this report, we have evaluated the effect of glycosylation on the autotransporter TibA. We have identified glycosylated peptides in TibA and found that the glycosylation was highly heterogenous. Some peptides were modified with various amounts of heptoses and, in some cases, a single peptide could be modified by up to seven heptoses. Unfortunately, in our study, we could not determine the precise sites of glycosylation. However, it is most likely to be O-glycosylation, as for AIDA-I and Ag43 [15], [25]. First, Aah, the glycosyltransferase associated with AIDA-I, is similar to TibC and, most likely, uses a similar mechanism of action [8]. In fact, the two glycosyltransferases are interchangeable [23]. Furthermore, most peptides do not contain motifs that are characteristic to N-glycosylation [37]. As in the case of AIDA-I and Ag43, we therefore hypothesize that the glycosylated residues are mainly serines, but could also be threonines [25].

TibA is heavily glycosylated compared to AIDA-I and could possess between 18 and 22 heptose residues. By comparison, 19 glycosylation sites were found in AIDA-I. However, the extracellular portion of AIDA-I, comprising the passenger domain and the junction region, is twice the length of that of TibA. In the case of AIDA-I, glycosylation was only found in the passenger domain [15]. In the case of Ag43, 16 glycosylation sites were found over the ∼500 first amino acids, until the position of the cleavage site in the passenger domain [25]. Unfortunately, in that study, the authors could not evaluate whether heptoses could be found beyond this point. From previous reports, it was therefore possible that the glycosylation of SAATs was specific of the imperfect repeats. Our results contradict that hypothesis and are in line with the recent observation that glycosylation is specific for a secondary structure and not a sequence [8]. Interestingly, only the ∼600 first amino acids of AIDA-I and TibA were glycosylated. We therefore hypothesize that the SAAT glycosyltransferases glycosylate the first 600 amino acids of the SAAT, independently of the domain to which they correspond and provided that they harbor the required secondary structure.

Taken together, our observations are consistent with the recently identified mechanism of substrate specificity for Aah [8]. Aah recognizes a structural motif consisting of a strand-loop-strand, with the glycosylated residue at the start of the loop. This rather loose mechanism of action probably applies to TibC and explains the glycosylation independently of any domain. Whether there is a mechanistical reason for the restriction to the first 500–600 amino acids is unclear and is under investigation.

We have also evaluated the effect of glycosylation on the functionality of TibA. We found that the glycosylation is essential for the adhesion properties of TibA, but not the other functions. This observation is in agreement with previous reports [24], [38] and also applies to AIDA-I [15]. This raised the possibility that glycosylation conferred lectin-like adhesive properties. However, in the case of AIDA-I, the protein is poorly expressed at the surface of the bacteria when not glycosylated. Therefore, the effect of glycosylation on the adhesion properties of AIDA-I could be an indirect effect caused by the low expression of unglycosylated AIDA-I. Unglycosylated TibA is expressed as well as glycosylated TibA, making it an ideal model to determine the exact effect of glycosylation on the functionality of SAATs. We observed that when unfolded, only purified glycosylated TibA could be refolded. We also observed that when not glycosylated, TibA is not sensitive to salt-induced oligomerisation and conformational changes whether we used purified proteins or proteins expressed in whole bacteria. Lastly, unglycosylated TibA caused a stress when expressed in *E. coli*. Taken together, our results suggest that the dynamics of TibA folding are affected by glycosylation. Thus, it seems that glycosylation gives flexibility to TibA, enabling conformational changes between an oligomeric autoaggregation-competent conformation and a monomeric adhesion-competent conformation. These observations made using a naturally glycosylated autotransporter that we can express in both its glycosylated and unglycosylated forms confirms previous studies performed with AIDA-I and Ag43 [15], [25]. Our observations confirm that the primary role of glycosylation is to ensure that the protein adopt a normal conformation, which indirectly affects the functionality of the protein. We therefore hypothesize that for all SAATs, glycosylation specifically affects conformation flexibility rather than allowing lectin-like interactions.
